# Genome-wide random regression analysis for parent-of-origin effects of body composition allometries in mouse

**DOI:** 10.1038/srep45191

**Published:** 2017-03-24

**Authors:** Jingli Zhao, Shuling Li, Lijuan Wang, Li Jiang, Runqing Yang, Yuehua Cui

**Affiliations:** 1 Key Laboratory of Aquatic Genomics, Ministry of Agriculture; Research Centre for Aquatic Biotechnology, Chinese Academy of Fishery Sciences, Beijing 100141, China; 2 Wuxi Fisheries College, Nanjing Agricultural University, Wuxi 214128, China; 3 College of Life Science, Northeast Agricultural University, Harbin 150030, China; 4 Key Laboratory of Experimental Marine Biology, Institute of Oceanology, Chinese Academy of Sciences, Qingdao 266071, China; 5 Department of Statistics and Probability, Michigan State University, East Lansing, MI 48864, USA; 6 Division of Health Statistics, School of Public Health, Shanxi Medical University, Taiyuan, 030001, China

## Abstract

Genomic imprinting underlying growth and development traits has been recognized, with a focus on the form of absolute or pure growth. However, little is known about the effect of genomic imprinting on relative growth. In this study, we proposed a random regression model to estimate genome-wide imprinting effects on the relative growth of multiple tissues and organs to body weight in mice. Joint static allometry scaling equation as sub-model is nested within the genetic effects of markers and polygenic effects caused by a pedigree. Both chromosome-wide and genome-wide statistical tests were conducted to identify imprinted quantitative trait nucleotides (QTNs) associated with relative growth of individual tissues and organs to body weight. Real data analysis showed that three of six analysed tissues and organs are significantly associated with body weight in terms of phenotypic relative growth. At the chromosome-wide level, a total 122 QTNs were associated with allometries of kidney, spleen and liver weights to body weight, 36 of which were imprinted with different imprinting fashions. Further, only two imprinted QTNs responsible for relative growth of spleen and liver were verified by genome-wide test. Our approach provides a general framework for statistical inference of genomic imprinting underlying allometry scaling in animals.

Genomic imprinting, an epigenetic phenomenon of parent-of-origin-specific gene expression, has been widely observed in plants^1^ and animals^2^,^3^,^4^,^5^,^6^,^7^,^8^,^9^ and has been recognized for its role in shaping developmental processes^10^,^11^,^12^. Genomic imprinting is a highly complex process that is involved in a number of growth axes operating coordinately at different development stages and showing a time-dependent effect during development. Most imprinted genes play important roles in controlling embryonic and post-natal growth and development in mammals. Based on the parental origin of the expressed allele, imprinting is classified into paternal imprinting and maternal imprinting. Imprinting can be further categorized as complete imprinting, when only one allele is expressed, or partial imprinting, when both alleles are expressed but at different levels^13^,^14^,^15^. To date, imprinting quantitative trait loci (iQTL) for growth and development traits have been identified in which the traits are measured in absolute growth. Little is known about how genomic imprinting affects relative growth, partially due to a lack of efficient statistical modelling and inference procedures.

As a measure of relative growth, allometry scaling describes the relationship between the entire body size and partial body size or between certain two biological traits. A “simple equation of allometry” has been initially developed to quantify allometry scaling^16^. In a simple allometry equation, the two variables include not only body size measured in scale of length and weight, but also body shape, density and volume. By taking into account the correlations among multiple body parts, the joint static allometry scaling model^17^ was proposed to simultaneously evaluate allometry scalings of multiple body parts to the entire body. Allometry scaling relationships between different biological traits contain three terms of allometries: static allometry, ontogenetic allometry and evolutionary allometry^18^,^19^,^20^. Static allometry refers to the relative growth between two different traits in adult or at a particular developmental stage. Ontogenetic allometry is the growth trajectory of one trait relative to the other in ontogeny. Evolutionary allometry is the relative growth between traits across species. The differentiation in allometries among traits has been thought to be a driving force by which morphology and structure evolve^21^.

Methods for detecting imprinting loci have been adapted from methods of interval mapping for Mendelian quantitative trait loci (QTL). Imprinting effects can be estimated using either least squares^9^,^22^,^23^,^24^ or maximum likelihood methods^25^. Multi-step tests for contrast models have been also proposed to identify the imprinting pattern^9^,^22^,^23^,^24^. With Bayesian model selection, Yang, *et al*.^26^ estimated genomic imprinting effects and inferred more genomic imprinting patterns than those summarized by Cheverud, *et al*.^27^. In the aspect of relative growth, genetic analyses for allometry scalings between biological traits have been carried out by embedding a simple allometry equation into additive genetic effects of the mixed linear model^28^,^29^ and into genotypic effects of genetic model for mapping QTL^30^,^31^,^32^. However, all these approaches to map allometry scalings are based on a single QTL model. When allometry scalings are controlled by multiple QTLs, they perform low power to detect QTLs. So far, no statistical method has been proposed to search for imprinted QTL for multiple allometry scalings.

Most current reports on genomic imprinting have been focused on brain and placenta in mammals^33^,^34^,^35^. Imprinting also affects adult traits either through the persistent effects of early growth and development^36^,^37^,^38^ or through direct effects on adult physiology. In absolute growth, a genome-wide mapping has been undertaken to identify quantitative trait nucleotides (QTNs) that have an imprinting effect on adult body composition using a three-generation intercross between inbred mouse strains^39^. To identify imprinted QTN for relative growth of tissues and organs to body weight in mice, here we developed a random regression model in which a joint static allometry scaling model is nested into genetic effects of markers and polygenic effects. The model was derived under the random regression framework, in which genetic effects of markers and genomic imprinting patterns are statistically inferred using chromosome-wide and genome-wide statistical tests.

## Methods

### Joint allometric scaling model

Let 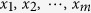 denote *m* partial body sizes and *y* be the entire body size. The joint static allometry model^17^ is defined as


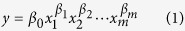


where *β*_0_ is an intercept, and 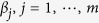 are partial scaling exponents of the *j*th partial component to the entire body size. Nonlinear least squares method is generally used to get unbiased estimators of the partial scaling exponents. Under the linear mixed model framework, a linear transformation of model (1) facilitates genetic analysis of allometric scalings. Taking the natural logarithm on both sides of model (1), we have


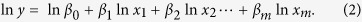


With the transformation, the joint static allometric scaling model can be optimized through a stepwise regression analysis.

### Random regression model for multiple static allometries

For a family-based population, suppose that *n* individuals are genotyped for *q* markers and observed for *m* partial body sizes. Four possible genotypes, denoted as QQ, Qq, qQ and qq, are distinguished at each marker. The first allele of each genotype is inherited from the paternal parent when considering the parental origin of alleles. In addition to the additive and dominance effects, the two reciprocal heterozygotes Qq and qQ carry the genetic imprinting effect that reflects the difference in allele expression derived from the two parents. Polygenic effects can also be estimated using data from multiple full-sibling and half-sibling families. Following a simple animal model for a single trait, the relationship between the logarithm of entire body size and markers can be modelled as


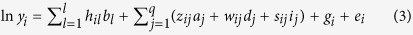


where *y*_*i*_ is the entire body size for the *i*th individual; *b*_*i*_ represents the *l*th fixed effect in *l* systematic environments, such as sexes; *a*_*j*_, *d*_*j*_ and *i*_*j*_ are additive, dominance and imprinting genetic effects for the *j*th marker; *h*_*il*_, *z*_*ij*_, *w*_*ij*_ and *s*_*ij*_ are indicator variables corresponding to *b*_*l*_, *a*_*j*_, *d*_*j*_ and *i*_*j*_, where *z*_*ij*_, *w*_*ij*_ and *s*_*ij*_ are defined in Mantey *et al*.^40^; *g*_*i*_ is the polygenic genetic effect derived from the pedigree information, assuming that 
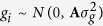
 with A being a relationship matrix and 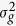 being the polygenic genetic variance; and *e*_*i*_ is the residual error with 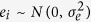.

To genetically analyse allometric scalings of multiple partial to entire body sizes, we embed model (2) into systematic environments, genetic effects of markers and polygenic effects in model (3), yielding the following random regression animal model^41^,^42^:


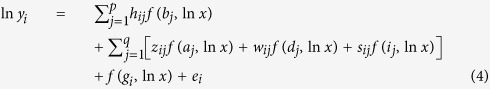


where 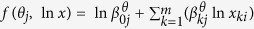 with *θ* ∈ *b, a, d, i* or *g*. Let 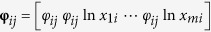, with **φ** ∈ **h**, **z**, **w** or **s** and *φ* ∈ *h, z, w* or *s*, 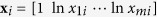, 
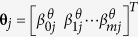
 with **θ** ∈ **b**, **a**, **d** or **i** and 
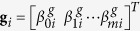
, then in matrix form, model (3) can be rewritten as,


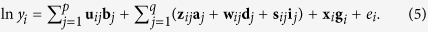


In this model, genetic effects are fixed for all markers and polygenic effects are random for all individuals. Thus,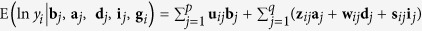 and 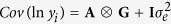, where **A** and **G** are the numerator-relationship matrix and polygenic genetic covariance matrix for multiple allometric scalings, respectively.

### Statistical inference for genomic imprinting

Restricted maximum likelihood method^43^ is implemented to estimate parameters in the random regression model described above, which gives maximum likelihood estimates and standard errors for fixed genetic effects at each marker, in addition to polygenic and residual variances. A student *t* statistic is formulated to statistically infer significance of marker genetic effect, which is calculated as


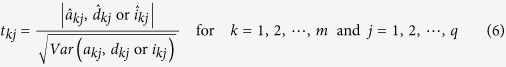


Similarly, another *t* statistic is calculated as


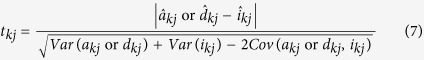


to statistically infer imprinting patterns.

The two test statistics asymptotically follow a standard normal distribution if the sample size is large enough so that the difference between the sample size and the number of estimated parameters exceeds 120. Under a standard normal distribution, the critical value is taken as 1.96 or 1.301 for −log(*p*) (*p* is the probability of the test statistic greater than 1.96) at the significance level of 5%. Thus, the iQTLs can be identified at chromosome-wide and genome-wide levels: by chromosome-wide test, the markers with significant genetic effects are screened from each chromosome, and then by genome-wide test, iQTLs are detected from all the significant markers screened by chromosome-wide tests.

In fact, we need to test whether or not a QTL exists, in which fashion the detected QTL inherits and what imprinting pattern the iQTL carries. A marker is identified as a QTL if one of the genetic effects is significantly different from zero by formula (6). In the case of no significant imprinting effect, the detected QTL is defined as a Mendelian QTL; otherwise, it is defined as an iQTL. The imprinting pattern can be further classified as either additive imprinting or dominance imprinting. Classification of imprinting patterns depends on the value and sign of *i* relative to *a* and *d*. The additive imprinting is composed of four subtypes: the complete or partial paternal additive imprinting corresponding to hypothesis *d* = 0 and *a* = *i* or *d* = 0 and *a* ≠ *i*, respectively; and the complete or partial maternal additive imprinting corresponding to hypothesis *d* = 0 and *a* = −*i* or *d* = 0 and *a* ≠ −*i*, respectively. The dominance imprinting is further classified into bipolar dominance (H_0_: *a* = 0 and *d* = 0), polar over-dominance (H_0_: *a* = 0 and *d* = *i*) and polar under-dominance (H_0_: *a* = 0 and *d* = −*i*). Following the definition of different imprinting types and the corresponding null hypothesis^26^,^27^,^44^, the imprinting pattern for the detected QTL can be statistically inferred by formula (7).

### Case analysis

A set of reciprocal cross families for distinguishing the four genotypes considering parental origin was derived from an F_2_ intercross of large (LG/J) and small (SM/J) inbred mouse strains^45^,^46^,^47^. Ten LG/J females were crossed with 10 SM/J males to produce 54 F_1_ hybrids. These F_1_ hybrids were intercrossed to produce 510 F_2_ animals. The F_2_ males and females were then reciprocally mated to produce 158 full-sibling F_2_ families with a total of 1,632 F_3_ progenies. Animals were sacrificed after 70 days of age. They were weighed to obtain an overall measure of body size (y). The length of the tail (*x*_1_) was measured with callipers. The mice were then immediately dissected by necropsy, and reproductive fat depot (*x*_2_), heart (*x*_3_), kidneys (*x*_4_), spleen (*x*_5_), and liver (*x*_6_) were weighed to the nearest 0.01 g with a digital scale.

A total of 353 single nucleotide polymorphism markers were chosen from the 4,200 polymorphic markers scored as part of the CTC/Oxford genotyping consortium. These makers were genotyped for all F_2_ animals and their F_3_ offspring. The F_2_ and F_3_ genotypes were used to reconstruct haplotypes using the “block-extension algorithm” in the PedPhase program^48^. With the inferred haplotype information, it was possible to distinguish all four genotypes in the F_3_ population at each marker locus with the paternal allele listed first and the maternal allele second.

Focusing on the form of absolute growth, Cheverud, *et al*.^27^ have mapped imprinting effects on the six tissues and organs in mice to know about the contribution of imprinting to quantitative variation in trait expression. By reanalysing the real dataset, we will estimate genomic imprinting effects and infer their patterns for relative growth of the adult body composition to body weight using the random regression model. Before gene mapping, these observations of traits were adjusted for the effects of ages at necropsy and litter sizes at birth^45^ and the residuals with population mean were used in the following analysis.

Stepwise regression analysis showed that not all six tissues and organs are significantly associated with body weight in relative growth. Thus, we dropped non-significant partial allometry exponents for model (2), and reached the following phenotypic joint static allometry scaling model by


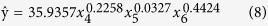


where *x*_4_, *x*_5_ and *x*_6_ refer to allometry scaling variables for kidney, spleen and liver, respectively. The joint static allometry scaling model of kidney, spleen and liver were chosen for a genome-wide random regression analysis to infer imprinting allometries; fatpad, tail and heart were excluded from the model due to their non-significant phenotypic partial allometry scalings effects.

The gender variable was considered as fixed in the final random regression model of multiple static allometries. Each fixed effect and marker genetic effects were estimated for the partial allometry exponents, along with the covariance matrix for polygenic effects and residual variance in the random regression model were estimated using REML via the DMU package. The initial values were defaulted as zero for each fixed effect, as identity matrix for the additive genetic covariance matrix and as one for the residual variance. Convergence precision for REML was set to 10^−6^.

**Figure 1:**
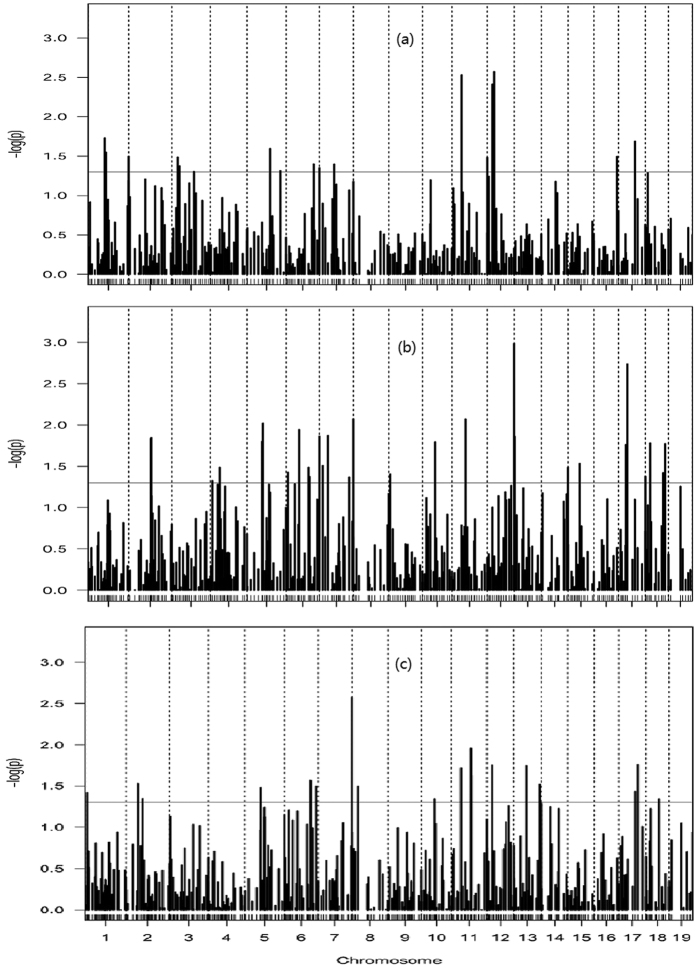
The profiles of test statistics of imprinted QTNs for relative growth of kidney (**a**), spleen (**b**) and liver (**c**) to body weight in mouse. The horizontal line in each plot represents the critical value of 1.301 for −log(*p*).

Results showed that a total of 122 QTNs were detected using the chromosome-wide tests. These QTNs were distributed on all chromosomes, and 48, 54 and 44 QTNs were associated with the relative growth of kidney, spleen and liver to body weight, respectively (results are shown in Table 1, Table 1S and Table 2S of the Supplementary file). Eleven QTNs were simultaneously associated with two of the three organs, showing pleiotropic effects. The profiles of test statistics for imprinting allometries are depicted in Fig. 1 for kidney (upper), spleen (middle) and liver (bottom). A total of 13, 15 and 11 markers were identified to be imprinted for relative weights of kidney, spleen and liver, respectively, because their test statistics exceeded the critical value of 1.301 at the 5% significance level, as displayed in Fig. 1. Using the chromosome-wide test, Table 1 tabulates the imprinted QTNs for relative growth of kidney, spleen and liver to body weight in mouse. For these imprinted QTNs, three show pleiotropy effects; that is, marker 103 (on chromosome 6) is associated with the relative weight of both spleen and liver, while marker 118 (on chromosome 8) and 206 (on chromosome 11) have effects on the relative weight of both kidney and liver. Genome-wide tests further verified that only 18 QTNs are inferred to be statistically significant at the 5% significance level, among which two are imprinted: marker 151 (on chromosome 15) regulates the relative weight of spleen and marker 184 (on chromosome 12) regulates the relative weight of liver.

Most of the imprinted QTNs inherit in bipolar dominance fashion with no significant additive and dominant effects. A total of 5 QTNs with over-dominance pattern control relative growths of three organs to body weight, among which SNPs rs3683086, rs13482635 and rs3713033 on chromosome 11, 15 and 19, respectively, were correlated with the kidney trait, while rs6296621 on chromosome 13 and rs3683086 on chromosome 11 were associated with spleen and liver traits, respectively. In particular, allometry of kidney to body weight was found to be regulated by imprinted SNP rs3688854 (on chromosome 2) in an under-dominance fashion, and allometry of spleen was regulated by SNP rs13480638 (on chromosome 10) with a complete maternal additive imprinting pattern. SNP rs13475748 (on chromosome 1) was inferred to be imprinted for the relative growth of liver to body weight, but its imprinting pattern was not defined since both the additive and dominant effects were significant.

Table 2 provides the significant QTNs for relative growth of kidney, spleen and liver to body weight based on the genome-wide test results. Eighteen out of 122 QTNs passed the genome-wide test, among which 4, 9 and 5 were responsible for kidney, spleen and liver, respectively. Some QTNs showed an additive effect on allometries of three organs but no dominance effect (e.g., on liver). Two imprinted QTNs, rs13482486 (on chromosome 15) and rs3662939 (on chromosome 12) on spleen and liver, respectively, were further verified through the genome-wide test. Among the significant QTNs and polygene effects by genome-wide test, the two imprinted QTNs contributed 17.12% and 37.12% of the total genetic variances for the relative growth of spleen and liver, respectively.

### Simulation study

The purpose of simulation was to investigate the statistical behaviour of detecting QTNs inherited in different patterns with the genome-wide random regression analysis. Based on the results from real data analysis, five additive/dominant QTNs (on chromosome 2) and two imprinting QTNs (on chromosomes 12 and 15) were chosen for simulation analysis. In the simulation, the phenotypic values for kidney, spleen and liver were retained as well as the genotypes of genetic markers. Logarithm of body weights was generated by the estimated fix regression effects for sex 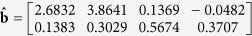, residual variance 
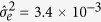
 obtained from the real data analysis, and polygenic genetic covariance matrix 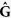 for multiple allometry scalings. Two simulation scenarios were considered. In scenario 1, we evaluated the precision of parameter estimation and the power of QTN detection by generating logarithm of body weights with 
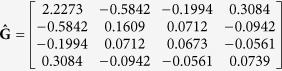
 estimated from the real data analysis. In scenario 2, we investigated the performance of QTN detection with 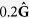. Simulations were repeated 100 times in each scenario to assess the power of QTN detection and precision of parameter estimation. Parameter estimates and statistical powers of QTL detection with genome-wide random regression analysis are shown in Table 3 for the simulated datasets. As can be seen, the higher the relative contribution of the simulated QTN, the greater the power to detect the QTN, which meets the general statistical behaviour in QTL mapping. When a QTN was detected, its genetic patterns can be further accurately inferred. In addition, we found that the power to detect the dominant and imprinting QTNs was low, as compared with that needed to identify additive QTNs. This observation is consistent with a regular QTL mapping study in which a QTL with an additive effect is easier to detect compared with the one with a dominant effect.

## Discussion

Taking the joint static allometry scaling model^49^ as sub-model, we constructed the random regression model to statistically infer genomic parent-of-origin effects on the relative growth of body composition to body weight in mice. With comparison to the random regression model for growth and developmental traits^42^ and mapping procedure for allometry scalings^30^,^31^,^32^, there are three major advantages of our analysis method. First, the joint static allometry scaling model can more accurately estimate allometry scalings of multiple tissues and organs to body weights than the simple allometry equation^16^. More importantly, it facilitates the comparison and genetic analysis of multiple allometry scalings. Second, when analysing growth and developmental traits with the random regression model, it is required for each individual to repeatedly measure the traits in growth and developmental duration. However, such repeat measurement is not a necessity in our study, because for each individual, body compositions were measured only once at necropsy. Third, our method incorporates polygenic effects derived from a pedigree into a random regression model, improving the estimation accuracy of marker effects.

It should be noted that our method can provide the estimates of iQTNs’ effects and polygenic effects but not heritabilities of iQTNs; that is, it cannot answer how much iQTNs contribute to phenotypic variation. If experimental individuals are sacrificed at different ages, the residual covariance matrix for multiple allometries can be estimated by nesting the joint static allometry model into permanent environmental effects caused by multiple ages at necropsy. A complete random regression model can therefore be constructed to identify genomic imprinting for joint allometries, denoted as


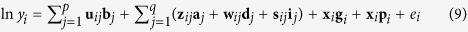


where **x**_*i*_**p**_*i*_ are random family and permanent environmental effect on multiple allometries. This allows us to successfully evaluate the genetic variation of multiple allometries, such as estimation of heritability for allometry scaling of each tissue and organ to body weight. In addition, a complete pedigree of many families and more recorded individuals are required to stably estimate the parameters in such a complex model. When separating real dataset with gender in case analysis, sub-dataset from male population is even not convergent in REML, this limits discussion about the effect of gender on imprinting status.

With the same dataset, Cheverud, *et al*.^27^ examined the contribution of imprinting to quantitative variation in trait expression by estimating imprinting effects on absolute growths of adult body composition traits. Of the eight pleiotropic iQTL the authors identified, only those on chromosomes 7, 12, and centromeric 18 were located in regions previously reported containing imprinted genes^50^. Their findings of imprinting loci, effects and patterns on adult body compositions were strongly supported by genetic evidences of imprinting on chromosomes 7^51^, 12^52^ and 18^53^. In our study, most iQTL for relative growth of body compositions are in new locations that have not previously been associated with imprinting effects on the absolute growth. Only six iQTLs for relative growth overlap with the four iQTLs identified for absolute growth on chromosomes 7, 12, and 18. In particular, the iQTLs for the relative growth on chromosomes 7, 12, and 18 have the same imprinting patterns as those for absolute growth, suggesting the existence of imprinting genes controlling both absolute and relative growth. By the chromosome-wide test, although the detected QTNs with additive, dominance or imprinting effect almost distributed on all chromosomes (see Tables 1S and 2S), only two iQTNs on chromosomes 12 and 15 were found to be associated with the relative growth of spleen and liver by the genome-wide test. Notably, the Pref-1/Dlk1 gene regulating growth retardation and accelerated adiposity is located on chromosome 12 in mice^54^.

If high density genetic markers are available that can distinguish the four genotypes, the method proposed here can be improved by doing an efficient marker selection, so that zero genetic effects can be first shrunk to zero by the least absolute shrinkage and selection operator^55^,^56^ for a sparse oversaturated regression model. Then, chromosome-wide or genome-wide non-zero genetic effects can be statistically inferred within the framework of the proposed model (5). If the detected QTNs only contribute to a small proportion of genetic variation for allometries, genome selection, for instance, whole genome regression^57^,^58^ and genomic best linear unbiased prediction^59^,^60^,^61^ can be introduced to assess genomic variation of allometries based on our proposed model with high density markers.

## Additional Information

**How to cite this article:** Zhao, J. *et al*. Genome-wide random regression analysis for parent-of-origin effects of body composition allometries in mouse. *Sci. Rep.* **7**, 45191; doi: 10.1038/srep45191 (2017).

**Publisher's note:** Springer Nature remains neutral with regard to jurisdictional claims in published maps and institutional affiliations.

## Supplementary Material

Supplementary Information

## Figures and Tables

**Table 1:** Chromosome-wide imprinted QTNs for relative growth of kidney, spleen and liver to body weight in mouse.

Organ	Chr.	QTN	Effect	Se	−log(p)	Imprinting pattern
Kidney	2	rs13476790	−0.246	0.108	1.641	Bipolar
	2	rs3688854	0.165	0.065	1.968	Under-dominance
	2	rs3715478	0.323	0.160	1.367	Bipolar
	7	CEL-7_116160192	0.213	0.105	1.376	Bipolar
	7	gnf07.120.460	−0.235	0.111	1.472	Bipolar
	8	rs13480023	−0.226	0.079	2.38	Bipolar
	8	rs3695597	0.174	0.076	1.643	Bipolar
	9	rs13480180	−0.177	0.077	1.669	Bipolar
	11	rs3683086	0.167	0.077	1.527	Over-dominance
	15	rs13482635	−0.165	0.074	1.596	Over-dominance
	19	rs3713033	−0.110	0.056	1.316	Over-dominance
Spleen	4	mCV24740485	−0.128	0.065	1.316	Bipolar
	6	rs13478681	−0.143	0.061	1.718	Bipolar
	9	rs6182207	0.134	0.063	1.462	Bipolar
	10	rs13480638	−0.218	0.078	2.263	Complete maternal additive
	10	rs13480797	−0.114	0.053	1.506	Bipolar
	10	rs3704401	−0.113	0.057	1.324	Bipolar
	10	rs6312070	0.151	0.063	1.793	Bipolar
	13	rs13481990	−0.159	0.045	3.401	Bipolar
	13	rs3718727	0.122	0.058	1.448	Bipolar
	13	rs6271232	0.106	0.049	1.497	Bipolar
	13	rs6296621	0.158	0.048	2.958	Over-dominance
	15	rs13482461	−0.186	0.070	2.078	Bipolar
	15	rs13482486	0.205	0.065	2.773	Bipolar
	18	gnf18.051.412	−0.140	0.070	1.351	Bipolar
	19	rs6307076	−0.091	0.046	1.314	Bipolar
Liver	1	rs13475748	−0.263	0.110	1.784	Undefined imprinting
	1	rs13475769	0.314	0.135	1.710	Bipolar
	3	rs13477364	0.211	0.089	1.751	Bipolar
	3	rs3658914	−0.261	0.095	2.215	Bipolar
	4	rs13478051	0.216	0.096	1.617	Bipolar
	6	rs13478681	0.258	0.114	1.631	Bipolar
	6	rs6339546	0.303	0.136	1.578	Bipolar
	8	rs13480023	0.166	0.079	1.454	Bipolar
	11	rs3683086	−0.162	0.076	1.492	Over-dominance
	12	rs3662939	−0.325	0.161	1.358	Bipolar
	12	rs3686891	0.339	0.158	1.490	Bipolar
	14	rs13482174	−0.266	0.100	2.091	Bipolar
	16	rs4170074	0.218	0.093	1.723	Bipolar

**Table 2:** Genome-wide QTNs for relative growth of kidney, spleen and liver to body weight in mouse.

Organ	Chr.	QTN	Inheritance	Effect	Se	−log(p)
Kidney	2	rs3688854	dominance	−0.180	0.061	2.500
	4	rs13477854	dominance	0.412	0.142	2.436
	12	rs13481408	additive	0.277	0.099	2.574
	16	rs4219239	additive	−0.173	0.081	1.497
Spleen	1	gnf01.157.188	dominance	0.126	0.063	1.351
	2	rs6252400	additive	0.323	0.132	1.836
	2	rs6185704	dominance	−0.113	0.053	1.481
	4	CEL-4_34055416	dominance	0.129	0.058	1.604
	6	rs13478974	additive	0.162	0.080	1.376
	7	rs3719258	dominance	−0.075	0.037	1.369
	11	CEL-11_118234030	dominance	−0.077	0.038	1.346
	15	rs13482486	imprinting	0.205	0.065	2.773
	18	gnf18.069.928	additive	0.117	0.056	1.422
Liver	2	rs13476473	additive	0.245	0.112	1.528
	2	rs3022886	additive	0.198	0.099	1.344
	6	rs3722157	additive	−0.223	0.101	1.566
	6	rs6199136	additive	−0.172	0.080	1.498
	12	rs3662939	imprinting	−0.325	0.161	1.358

**Table 3:** Parameter estimates (standard deviations) and statistical powers of QTL detection with genome-wide random regression analysis for simulated datasets.

Polygene	QTL	dQTL_1_	aQTL_2_	dQTL_3_	aQTL_4_	aQTL_5_	iQTL_6_	iQTL_7_
True	Chr.	2	2	2	2	2	12	15
	SNP	rs3688854	rs13476473	rs3022886	rs6252400	rs6185704	rs3662939	rs13482486
	Organ	Kidney	Liver	Liver	Spleen	Spleen	Liver	Spleen
	Effect	−0.180	0.245	0.198	0.323	−0.113	−0.325	0.205
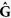	Effect	−0.161(0.09)	0.227(0.08)	0.164(0.07)	0.303(0.06)	−0.098(0.08)	−0.301(0.06)	0.182(0.09)
	Power	54%	72%	59%	84%	56%	70%	62%
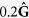	Effect	−0.179(0.07)	0.238(0.06)	0.183(0.05)	0.328(0.07)	−0.128(0.07)	−0.333(0.06)	0.209(0.07)
	Power	75%	96%	78%	100%	76%	91%	82%
