# Supplementary material for: Genome-wide random regression analysis for parent-of-origin effects of body composition allometries in mouse
Source: Sci Rep. 2017 Mar 24;7:45191. doi: 10.1038/srep45191 (PMC5364555; doi:10.1038/srep45191)
Supplement: Supplementary Information [file srep45191-s1.pdf]

Genome-wide random regression analysis for parent-of-origin effects of body composition allometries in Mouse

Jingli Zhao[#1,2], Shuling Li[#3], Lijuan Wang[4], Li Jiang[2],
Runqing Yang*[1] and Yuehua Cui*[5,6]

1. *Key Laboratory of Aquatic Genomics, Ministry of Agriculture; Research Centre for Aquatic Biotechnology, Chinese Academy of Fishery Sciences, Beijing 100141, China*
2. *Wuxi Fisheries College, Nanjing Agricultural University, Wuxi 214128, China*
3. *College of Life Science, Northeast Agricultural University, Harbin 150030, China*
4. *Key Laboratory of Experimental Marine Biology, Institute of Oceanology, Chinese Academy of Sciences, Qingdao 266071, China*
5. *Department of Statistics and Probability, Michigan State University, East Lansing, MI 48864, USA*
6. *Division of Health Statistics, School of Public Health, Shanxi Medical University, Taiyuan, 030001, China*

Running Head: Imprinting for body composition allometries

Submitted to 'SREP'

[#] These authors contributed equally to this study.
*Author for correspondence:

Runqing Yang, PhD
Research Centre for Aquatic Biotechnology
Chinese Academy of Fishery Sciences
Beijing 100141, People's Republic of China
Tel: +86-10-68670701
Fax: +86-10-68670701
E-mail: runqingyang@cafs.ac.cn

Yuehua Cui, PhD
Department of Statistics and Probability
Michigan State University
East Lansing, MI 48864, USA
E-mail: cui@stt.msu.edu

Table 1S Genome-wide additive QTNs for relative growth of kidney and spleen to body weight in mouse

Organ	Chr.	SNP	Imprinted	Effect	Se	Log(p)
Kidney	1	rs13475945		-0.227	0.110	1.419
	1	petM22381-169-2		-0.279	0.118	1.730
	2	rs13476319		0.303	0.138	1.549
	3	rs4223864		-0.170	0.079	1.499
	3	gnf03.036.164		0.245	0.112	1.528
	3	gnf03.117.090		0.198	0.099	1.344
	5	rs3658755		0.323	0.132	1.836
	5	rs6393330		-0.329	0.134	1.848
	6	rs13479024		0.206	0.096	1.487
	6	rs6265387		-0.240	0.118	1.376
	7	rs13479342		-0.222	0.113	1.305
	11	rs3690160		-0.114	0.057	1.328
	12	rs3699421		-0.124	0.058	1.488
	12	rs6223000		-0.156	0.065	1.801
	12	rs13481408		-0.243	0.114	1.481
	16	rs4219239		0.187	0.072	2.023
	17	rs6210797		-0.252	0.113	1.596
Spleen	2	rs6252400		0.137	0.069	1.317
	2	rs6406705		-0.157	0.076	1.428
	4	rs13477576		0.248	0.098	1.945
	4	rs3663355		-0.136	0.064	1.487
	5	rs3711950		0.162	0.080	1.376
	5	rs3691938		-0.223	0.101	1.566
	6	rs13478645		-0.262	0.127	1.399
	6	rs8270116		-0.172	0.080	1.498
	6	rs13478949		-0.130	0.065	1.351
	6	rs13478974		0.079	0.032	1.865
	7	CEL-7_12787527		-0.093	0.043	1.511
	7	rs13479174		-0.107	0.043	1.872
	7	rs3719256		-0.178	0.087	1.397
	7	rs3719258		-0.077	0.038	1.368
	7	rs6216320		0.146	0.055	2.075
	9	gnf09.009.970		-0.192	0.064	2.579
	10	rs13480638	Yes	0.138	0.064	1.498
	11	rs13481031		0.115	0.056	1.406
	13	rs6215262		0.188	0.078	1.795
	13	rs13481689		-0.254	0.127	1.342
	14	rs13482416		0.326	0.110	2.530
	15	rs4139476		-0.251	0.107	1.720
	17	CEL-17_40073719		-0.166	0.063	2.073

	17	rs13483011		-0.402	0.158	1.960
	18	rs13483183		0.343	0.151	1.636
	18	rs3722205		-0.177	0.083	1.484
	18	gnf18.069.928		0.231	0.097	1.758
	18	rs3705890		-0.246	0.085	2.413
Liver	1	rs13475748	Yes	0.277	0.092	2.574
	2	rs13476473		-0.349	0.106	2.986
	2	rs3022886		0.288	0.117	1.863
	5	rs3691938		0.226	0.095	1.753
	6	rs3722157		0.224	0.104	1.517
	6	rs6199136		0.119	0.055	1.489
	8	rs6273176		-0.146	0.067	1.535
	8	CEL-8_25677705		-0.173	0.081	1.497
	10	rs13480638	Yes	0.215	0.090	1.762
	11	rs3690160		-0.138	0.044	2.739
	11	rs13481093		-0.168	0.081	1.437
	11	rs6381209		0.171	0.074	1.691
	12	rs6223000		0.166	0.070	1.765
	13	rs3718727	Yes	0.135	0.066	1.380
	13	rs13482028		0.161	0.067	1.783
	17	rs6210797		-0.135	0.067	1.342
	17	rs13483110		0.117	0.056	1.422
	18	CEL-18_60214752		-0.124	0.052	1.774

Table 2S Genome-wide dominant QTNs for relative growth of spleen and liver to body weight in mouse

Organ	Chr.	SNP	Imprinted	Effect	Se	Logp
Kidney	1	gnf01.117.970		0.271	0.105	2.016
	2	rs3688854	Yes	0.381	0.190	1.349
	4	rs13477831		0.193	0.077	1.907
	4	rs13477854		-0.272	0.118	1.665
	4	rs3720634		0.126	0.063	1.351
	6	rs13478645		-0.180	0.061	2.500
	8	CEL-8_25677705		-0.169	0.084	1.365
	8	rs3672639		-0.223	0.101	1.551
	8	rs13479811		0.469	0.154	2.638
	9	rs3670579		-0.279	0.120	1.711
	11	rs3683086	Yes	0.225	0.112	1.353
	12	rs4229612		-0.113	0.053	1.481
	12	rs3679276		0.129	0.058	1.604
	13	rs4229685		-0.303	0.114	2.112
	15	rs13482461	Yes	0.155	0.060	2.019
	15	rs3692040		-0.351	0.152	1.684
	15	rs6400804		0.412	0.142	2.436
	15	rs13482635	Yes	0.139	0.058	1.769
	16	rs4170074	Yes	-0.341	0.124	2.218
	16	rs4174469		-0.329	0.123	2.113
	17	rs3724616		0.363	0.122	2.550
	18	rs13483226		0.151	0.068	1.561
	19	rs3713033	Yes	0.123	0.056	1.540
	19	rs6307076	Yes	-0.075	0.037	1.369
Spleen	1	rs6250257		0.133	0.060	1.598
	1	rs3688042		-0.127	0.059	1.500
	1	gnf01.157.188		0.142	0.066	1.496
	2	rs6185704		0.388	0.161	1.795
	4	CEL-4_34055416		-0.399	0.171	1.709
	4	rs3671277		0.314	0.146	1.509
	6	rs6339546	Yes	-0.277	0.122	1.640
	7	gnf07.120.460	Yes	0.173	0.071	1.841
	7	rs3719258		0.187	0.094	1.339
	7	rs6299045		-0.204	0.101	1.368
	11	CEL-11_118234030		0.153	0.073	1.455
	13	rs6296621	Yes	-0.178	0.076	1.723
	13	rs13481947		-0.077	0.038	1.346
	14	rs13482206		-0.367	0.144	1.970
Liver	1	rs13475748	Yes	0.338	0.134	1.939
	2	rs3718711		-0.158	0.079	1.346
	2	rs3713848		-0.232	0.093	1.879

2	mCV23002990		0.111	0.051	1.556
2	rs3681694		-0.106	0.048	1.556
2	rs13476790	Yes	-0.104	0.048	1.531
4	rs3671277		0.198	0.077	1.978
6	CEL-6_10519419		0.255	0.127	1.342
6	rs13478645		-0.242	0.106	1.650
8	CEL-8_25677705		0.225	0.114	1.313
8	rs3672639		-0.186	0.072	1.999
8	rs13479811		-0.179	0.081	1.555
9	rs13480065		0.215	0.081	2.082
9	rs3670579		0.165	0.067	1.881
11	rs3683086	Yes	-0.231	0.117	1.310
13	rs6296621	Yes	-0.143	0.058	1.858
14	rs13482206		0.143	0.062	1.667

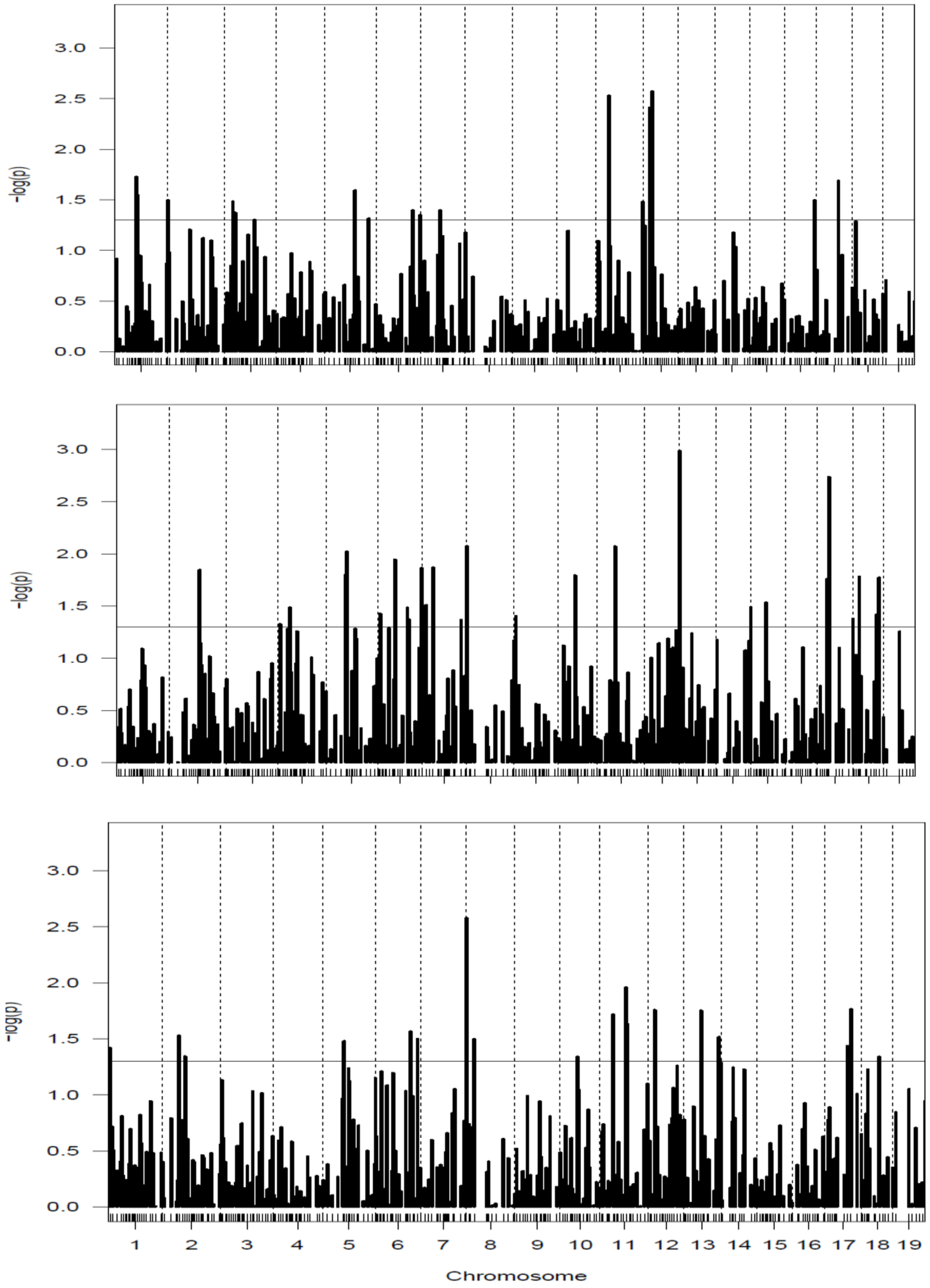

Figure 1S The profiles of test statistics for additive effects on relative growths of kidney (Upper), spleen (Middle) and liver (Bottom) to body weight in mouse

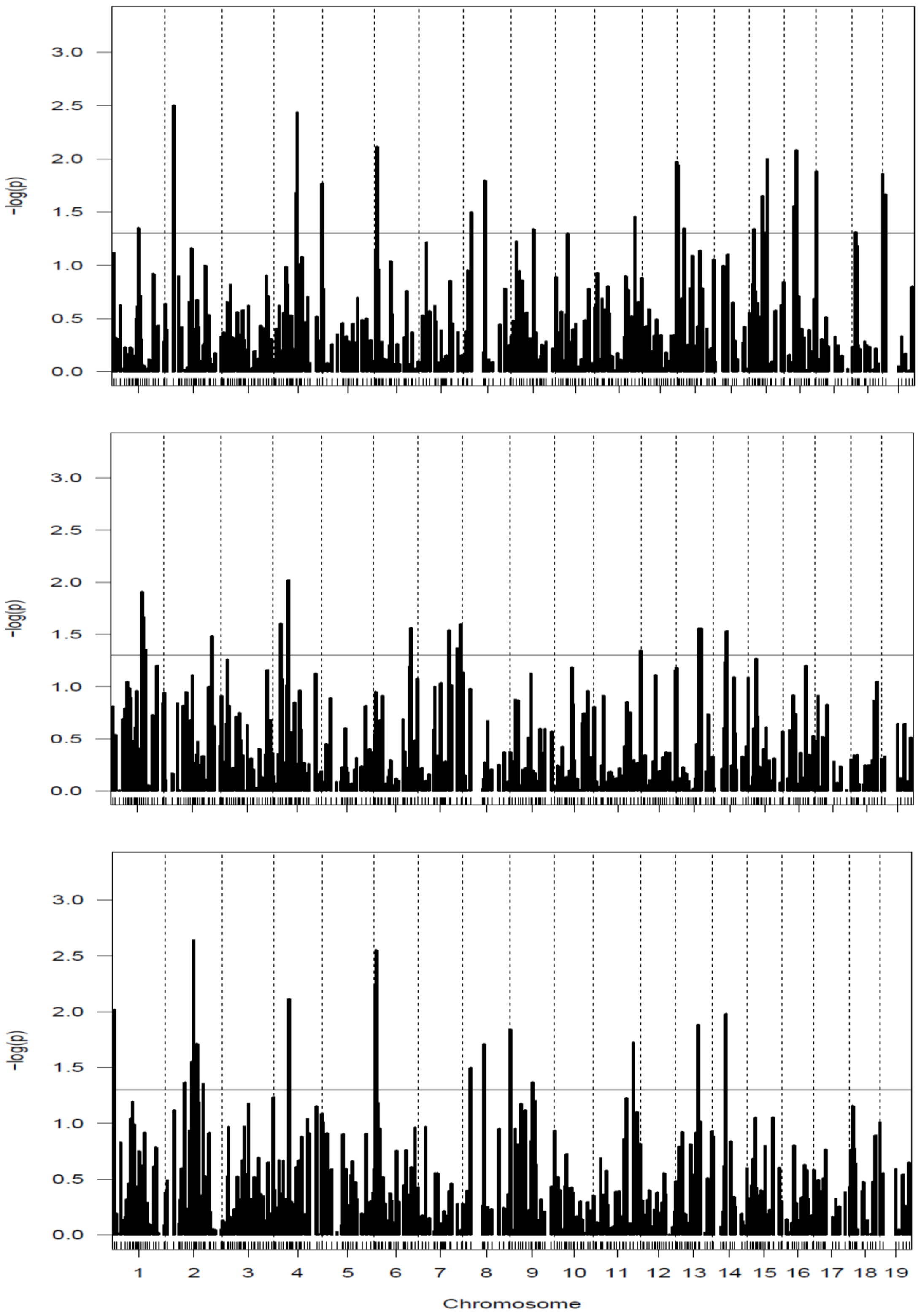

Figure 2S The profiles of test statistics for dominant effects on relative growths of kidney (Upper), spleen (Middle) and liver (Bottom) to body weight in mouse
